# Metformin enhances alpelisib sensitivity in HER2+ breast cancer by suppressing cancer stemness and oncogenic signaling

**DOI:** 10.3389/fonc.2025.1631415

**Published:** 2025-11-19

**Authors:** Yujie Shi, Peijia Niu, Qiong Cheng, Lexia Chen, Yahan Weng, Xiaohe Yang

**Affiliations:** 1 Department of Pathology, People’s Hospital of Zhengzhou University, Zhengzhou, Henan, China; 2 Department of Pathology, Fuwai Central China Cardiovascular Hospital, Zhengzhou, Henan, China; 3 Department of Biological and Biomedical Sciences, North Carolina Research Campus, Biomedical/Biotechnology Research Institute, North Carolina Central University, Kannapolis, NC, United States

**Keywords:** alpelisib, metformin, HER2/ERBB2, cancer stem cells, synergy

## Abstract

**Introduction:**

Alpelisib, a selective PI3Kα inhibitor that targets PIK3CA mutations, is approved for hormone receptor–positive, HER2-negative breast cancer. However, its therapeutic potential in HER2-positive disease remains underexplored. Hyperglycemia, a frequent adverse event of alpelisib, limits its clinical application. Metformin, an antidiabetic drug with reported anticancer activity, may counteract alpelisib-induced hyperglycemia while enhancing its antitumor efficacy.

**Methods:**

We evaluated the effects of alpelisib alone and in combination with metformin in HER2-overexpressing breast cancer cell lines SK-BR-3 and BT-474. Cell viability, colony formation, and tumorsphere assays were performed to assess proliferation and stemness. Flow cytometry was used to analyze ALDH1-positive cell populations and cell-cycle distribution. Synergy was determined using the combination index method. Western blotting examined the effects on signaling pathways and stemness-associated proteins.

**Results:**

Alpelisib monotherapy inhibited proliferation, colony formation, and cancer stem cell features in both cell lines. Metformin enhanced these effects, demonstrating strong synergism. Combination treatment induced significant G0/G1 cell-cycle arrest, reduced ALDH1⁺ populations, and decreased tumorsphere formation. Mechanistically, co-treatment broadly suppressed receptor tyrosine kinase–mediated signaling, augmenting inhibition of PI3K/Akt/mTOR, MAPK/ERK, and JAK/STAT pathways, along with downregulation of c-Myc. Stemness-related proteins including β-catenin, Nanog, Sox2, KLF4, and LGR5 were also markedly reduced.

**Discussion:**

These findings indicate that metformin synergistically enhances the antitumor activity of alpelisib in HER2-positive breast cancer by inhibiting oncogenic signaling and stemness pathways. Beyond its metabolic benefit in mitigating hyperglycemia, metformin may potentiate PI3K-targeted therapies, supporting further preclinical and clinical evaluation of this combination strategy.

## Introduction

Breast cancer remains a significant clinical challenge due to its inherent complexity and heterogeneity. The development of targeted therapies has provided new opportunities to improve patient outcomes, with the phosphatidylinositol-3-kinase (PI3K) pathway emerging as a critical target in breast cancer management (1). Dysregulation of this pathway is implicated in tumorigenesis, disease progression, and metastasis (2, 3). Alpelisib, a selective inhibitor of the p110α subunit of PI3K (3), has demonstrated substantial clinical efficacy in hormone receptor (HR)-positive, HER2/erbB2-negative breast cancers harboring PIK3CA mutations (4–6). Its combination with endocrine therapy, such as fulvestrant, represents a significant advancement for patients’ resistant to prior endocrine therapies, establishing alpelisib as a key component of breast cancer treatment paradigms (7, 8).

Despite its therapeutic potential, the clinical application of alpelisib is hindered by notable challenges, including treatment-induced hyperglycemia, which can lead to severe complications such as ketoacidosis (9–11). This adverse effect is particularly concerning in patients with preexisting metabolic conditions, such as diabetes or insulin resistance, thereby limiting the broader application of alpelisib (10, 12). Furthermore, the heterogeneity of breast cancer and the emergence of resistance to alpelisib underscore the need for innovative strategies to enhance its efficacy and mitigate its limitations (13).

While alpelisib is primarily approved for HR+/HER2− breast cancer (5), there is increasing interest in its application in HER2-positive breast cancer (14–16). HER2 overexpression is frequently associated with hyperactivation of the PI3K signaling pathway (17), which presents a promising target for alpelisib. However, the complex signaling interactions in HER2-positive breast cancer may limit alpelisib’s efficacy (18), highlighting the necessity of combination therapies to optimize outcomes in this subgroup.

Metformin, a widely used antidiabetic drug, has garnered attention for its anticancer properties, including its ability to inhibit cancer cell proliferation and enhance the efficacy of conventional chemotherapies (19–21). Metformin exerts its effects through multiple mechanisms, such as activation of AMP-activated protein kinase (AMPK), suppression of mTOR signaling, and modulation of glucose metabolism (22). Importantly, by lowering systemic insulin levels, metformin targets a critical driver of tumor growth in insulin-responsive cancers (23). Preclinical studies have demonstrated that metformin, when combined with other therapeutic agents, can achieve greater tumor suppression and prolonged remission compared to monotherapies (24), suggesting its potential as a valuable adjunct in breast cancer treatment.

The hyperglycemic side effects of alpelisib pose a unique opportunity for combination therapy with metformin (10). In addition to counteracting alpelisib-induced hyperglycemia, metformin may potentiate its anticancer effects by further suppressing PI3K/mTOR signaling and targeting cancer stem cell populations (25, 26). Metformin’s potential effect on both hyperglycemia control and tumor inhibition supports the rationale for investigating the combination of alpelisib and metformin as a synergistic therapeutic strategy for breast cancer. Although recent clinical trials and studies (10, 11, 27) have reported the inclusion of metformin in alpelisib treatment, the primary focus has been on managing alpelisib-associated hyperglycemia. Therefore, it is crucial to investigate the effects and mechanisms of the alpelisib-metformin combination in inhibiting breast cancer cell tumorigenesis.

In this study, we explored the combined effects of alpelisib and metformin in HER2-positive breast cancer cell lines. Our findings demonstrate that alpelisib effectively suppresses cell proliferation and cancer stem cell traits in this context. Notably, the combination of alpelisib and metformin exhibited enhanced efficacy, characterized by greater inhibition of the PI3K and mTOR signaling pathways. These results provide a foundation for future clinical studies aimed at optimizing therapeutic regimens incorporating alpelisib and metformin, with the goal of improving outcomes for breast cancer patients.

## Materials and methods

### Reagents and antibodies

Alpelisib and metformin were ordered from LC Laboratories (Woburn, MA, USA) and Sigma-Aldrich (St Louis, MO, USA) respectively. Primary antibodies against phosphorylated ERK1/2 (Thr202/Tyr204) (p-ERK1/2, Cat: 9101), ERK1/2(Cat: 9102), phosphorylated Akt (Ser473) (p-Akt, Cat: 4060), Akt(Cat: 9272), phosphorylated Stat3 (Tyr705) (p-Stat3, Cat: 9145), Stat3 (Cat: 4904), Cyclin D1(Cat: 55506), c-Myc (Cat: 9402), phosphorylated ErbB2 (Tyr1221/1222) (p-ErbB2, Cat: 2243), ErbB2 (Cat: 2242), β-Catenin (Cat: 8480), Active β-Catenin (Cat: 19807), phosphorylated mTOR (Ser2448) (p-mTOR, Cat: 5536), mTOR (Cat: 2983), phosphorylated S6K (Thr389) (p-S6K, Cat: 9234), S6K (Cat: 9202), Nanog (Cat: 4903), KLF4 (Cat: 12173), Sox2 (Cat: 23064), AMPK (Cat: 2532), p-AMPK (Cat: 2535) along with Anti-mouse/rabbit HRP-linked secondary antibodies, were purchased from Cell Signaling Technology (Danvers, MA, USA). The antibodies against GAPDH (Cat: sc-47724) and LGR5 (Cat: sc-517295) were from Santa Cruz Biotechnology (Santa Cruz, CA, USA).

### Cell culture

The SK-BR-3 and BT-474 breast cancer cell lines were obtained from the American Type Culture Collection (ATCC) (Manassas, VA, USA). The cells were cultured in DMEM/F-12 medium supplemented with 10% fetal bovine serum (FBS), 100 μg/mL penicillin, and 100 μg/mL streptomycin. They were maintained in a humidified incubator at 37 °C with 5% CO2.

### Cell proliferation assay

Cell Proliferation was determined by Cell Counting Kit-8 (CCK-8) assay. The cells were seeded into 96-well plates (1 × 10^3^ cells/well) for 24 h and then treated with metformin or alpelisib, or in combination, at the indicated concentrations for 5 days. The 5-day time point was selected based on preliminary studies and established protocols to capture the sustained anti-proliferative effects of the treatments. At each endpoint, 10 μl of CCK-8 reagent was added into each well and was then incubated for two hours at 37 °C. The colorimetric absorbance at 450 nm was measured with an ELISA microplate reader. Six parallel replicates per experiment were analyzed for each experimental sample. Representative data from three repeats were presented.

### Clonogenic assays

Cells were plated (600 cells/well) in 6-well plates for 24 h, followed by treatment with alpelisib or metformin at indicated concentrations for 14 days. Then, the colonies were fixed with 4% paraformaldehyde and stained with 0.5% crystal violet. Colonies were counted using ImageJ software. Representative data from three sets of repeats were presented.

### Cell cycle analysis with flow cytometry

The treated cells from each group were collected by trypsinization, and the resulting single-cell suspensions were fixed in 70% ethanol overnight at −20 °C. The fixed cells were washed with PBS and incubated for 30 minutes at 37 °C in a solution containing 0.05% Triton X-100 in PBS, RNase A (100 μg/mL), and propidium iodide (PI, 50 µg/mL). Cell cycle phase distribution was analyzed using a CytoFLEX flow cytometer (Beckman Coulter, Brea, CA, USA). The percentages of cells in each phase were determined using ModFit software. Representative data from three independent experiments are presented.

### Tumorsphere assays

Cells were seeded into ultra-low attachment 24-well plates (Corning) at a density of 1, 000 cells per well. The cells were treated with the indicated doses of alpelisib, metformin, or their combination and incubated in DMEM/F-12 medium supplemented with 10 μg/mL insulin (Sigma, St. Louis, MO, USA), 1 μg/mL hydrocortisone (Sigma), 1× B27 (Thermo Fisher Scientific, Waltham, MA, USA), 20 ng/mL EGF (Stemcell Technologies, Vancouver, BC, Canada), 20 ng/mL bFGF (Stemcell Technologies), and 4 μg/mL heparin (Stemcell Technologies) for seven days to assess primary sphere formation. Spheres with diameters >25 μm were counted and imaged. For secondary sphere formation, single-cell suspensions, generated from the primary spheres using trypsinization, were replated under the same conditions to evaluate secondary sphere formation. Sphere counting and imaging were performed following the same protocol. Primary and secondary sphere formation was analyzed in triplicate.

### ALDEFLUOR assay

The ALDEFLUOR assay kit (STEMCELL Technologies, Vancouver, Canada) was used to determine the percentage of ALDH1+ cells in SK-BR-3 cells following treatment. Cells were trypsinized and resuspended in assay buffer containing the ALDH substrate according to the manufacturer’s instructions. Negative controls were prepared by incubating a portion of the sample with the ALDH inhibitor, diethylaminobenzaldehyde (DEAB). All samples were incubated at 37 °C for 45 minutes, washed, and resuspended in assay buffer. ALDH activity was analyzed using a CytoFLEX flow cytometer, and data were processed with CytExpert software. The percentage of ALDH1-positive cells was quantified based on gating established with DEAB-treated controls.

### Western blot analysis and quantification

SK-BR-3 and BT-474 cells were treated with alpelisib, metformin, or their combination at the indicated concentrations for 48 hours. The treatment duration was selected to capture peak, steady-state modulation of signaling pathway activity following drug exposure, particularly to metformin. With the cell lysate prepared from the treated cells, protein concentrations were determined using a BCA Protein Assay Kit (Thermo Scientific, Rockford, IL, USA), and equal amounts of protein (50 µg) were resolved on 8–15% SDS-PAGE gels before being transferred onto nitrocellulose membranes. The membranes were blocked with 5% non-fat milk in TBST for 2 hours at room temperature and incubated with specific primary antibodies overnight at 4 °C. After washing with TBST, the membranes were incubated with horseradish peroxidase (HRP)-conjugated secondary antibodies for 2 hours at room temperature. Following final washes in TBST, protein bands were visualized using enhanced chemiluminescence (ECL) reagents (Thermo Scientific) and imaged with a FluorChemE imager. Western blot band intensities were analyzed using ImageJ software. The integrated density of each target band was measured, background-corrected, and normalized to its corresponding loading control. For phosphorylated proteins, levels were further normalized to their corresponding total protein levels to evaluate the degree of activation/phosphorylation. Data from three independent experiments are presented as fold-change relative to the control.

### Drug synergy analysis

The synergistic effect between alpelisib and metformin was evaluated using CCK-8 assays as previously described (28). Cells were treated with alpelisib, metformin, or their combinations at the indicated concentrations for 5 days, followed by incubation with the CCK-8 reagent. Combination index (CI) values were calculated using the Chou–Talalay method with CompuSyn software (ComboSyn, Inc.; Paramus, NJ, USA) (29, 30). CI values of < 1, = 1, and > 1 represent synergistic, additive, and antagonistic effects, respectively.

### 3D culture for anchorage-independent growth

A base layer was prepared by adding 100 µL of 5% Matrigel in complete media (on ice) to each well of a 48-well plate and allowing it to polymerize for 30 minutes at 37 °C. Subsequently, cells were suspended in pre-cooled DMEM/F12 media supplemented with 10% FBS, containing 2X the indicated concentrations of Alpelisib, Metformin, their combination or control, at a density of 8, 000 cells/mL. An equal volume of this cell suspension was mixed with 5% Matrigel on ice to generate the working cell solution. Then, 300 µL of this mixture was carefully layered atop the pre-formed base layer. The plates were incubated for 14 days in a humidified 37 °C incubator with 5% CO_2_. After the incubation period, colonies were imaged, and those ≥25 µm in diameter were counted. Colony counts from triplicate wells were statistically analyzed using one-way ANOVA with Tukey’s *post-hoc* test.

### Statistical analysis

Statistical analyses, including the determination of significance and IC_50_ values, were performed using Prism 7 software (GraphPad, La Jolla, CA, USA). Statistical significance for data based on biological replicates from clonogenic, tumorsphere, and ALDEFLUOR assays was determined by one-way ANOVA followed by Tukey’s *post-hoc* test for multiple comparisons. Statistical significance was indicated as follows: * p < 0.05; ** p < 0.01.

## Results

### Alpelisib inhibits the proliferation and colony formation of erbB2-overexpressing breast cancer cells

**Figure 1:**
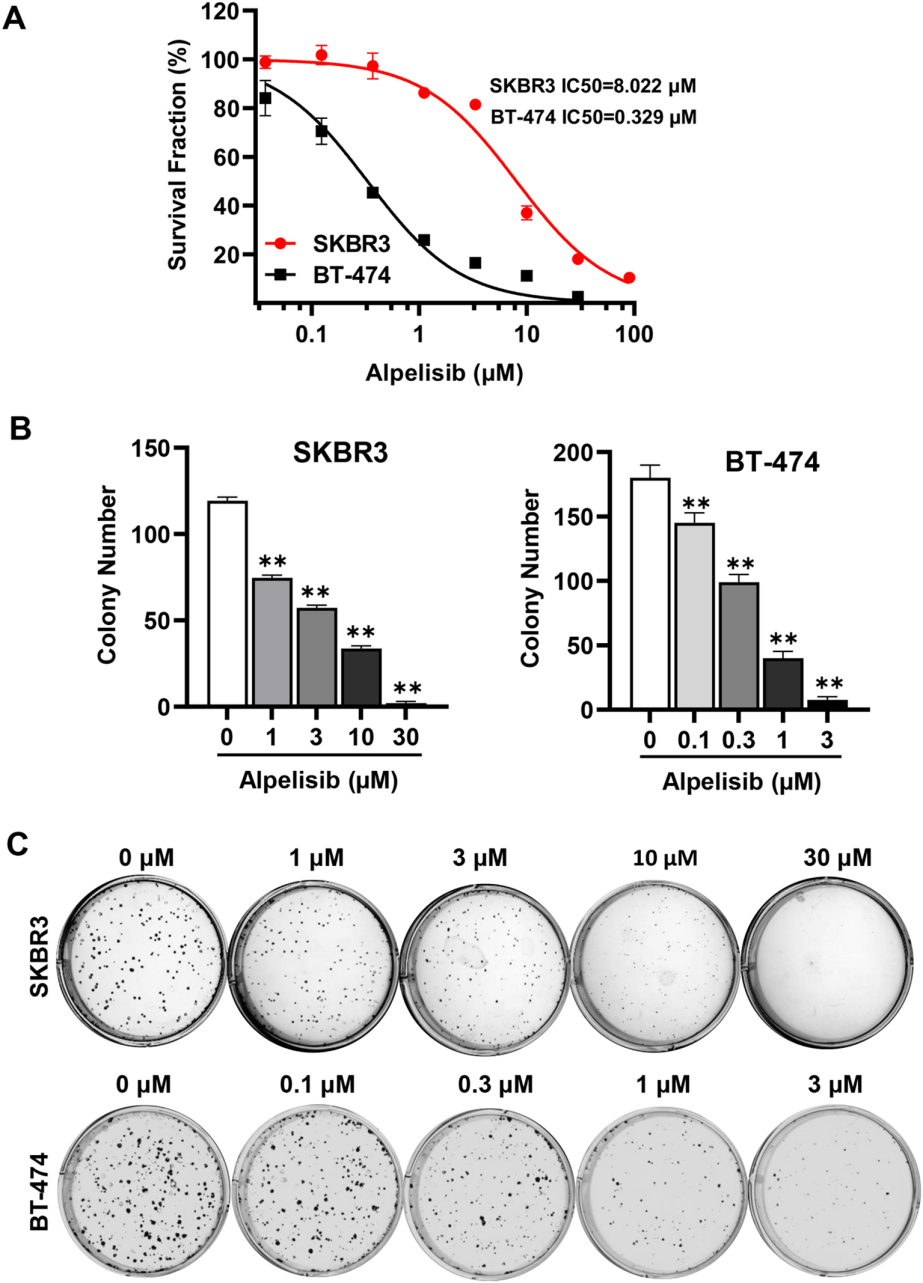
Alpelisib inhibits the proliferation and clonogenic potential of SK-BR-3 and BT-474 cells. **(A)** Alpelisib inhibits SK-BR-3 and BT-474 cell proliferation. The cells were treated with various concentrations of alpelisib (0, 0.1, 0.3, 1, 3, 10, 30, 100 μM) for 5 days, followed by CCK-8 assays. The IC_50_ values were calculated using GraphPad Prism software. **(B)** Alpelisib inhibits clonogenic formation of SK-BR-3 and BT-474 cells. The cells were seeded at 600 cells/well in 6-well dishes and treated with alpelisib at the indicated concentrations for 14 days. Following treatment, cells were fixed and stained with crystal violet. The data were analyzed by one-way ANOVA with Tukey’s test. for multiple comparisons. ** p < 0.01 (indicated group vs. the untreated control). **(C)** Representative images of clonogenic assays quantified in B.

We first evaluated the effect of alpelisib as a single agent on the growth of SK-BR-3 and BT-474 breast cancer cells, both of which overexpress erbB2. Using a CCK-8 assay, we measured cell proliferation after 5 days of alpelisib treatment. As shown in **Figure 1A**, alpelisib inhibited cell viability in a dose-dependent manner, with BT-474 cells showing greater sensitivity (IC_50_ = 0.329 μM) compared to SK-BR-3 cells (IC_50_ = 8.022 μM). The results indicate that alpelisib inhibits the proliferation of both cell lines, with BT-474 cells showing greater sensitivity than SK-BR-3 cells. To further assess alpelisib-induced tumor inhibition effects, we performed colony formation assays. The results likewise demonstrated significant suppression in both lines (**Figure 1B, C**). BT-474 cells again exhibited greater sensitivity than SK-BR-3 cells, with effective inhibition observed at concentrations of 0.1–3 μM for BT-474 and 1–10 μM for SK-BR-3. Taken together, these findings demonstrate that alpelisib effectively inhibits proliferation and clonogenic potential in erbB2-overexpressing breast cancer cells, underscoring its therapeutic potential in this subtype.

### Alpelisib inhibits stemness in erbB2-overexpressing breast cancer cells

**Figure 2:**
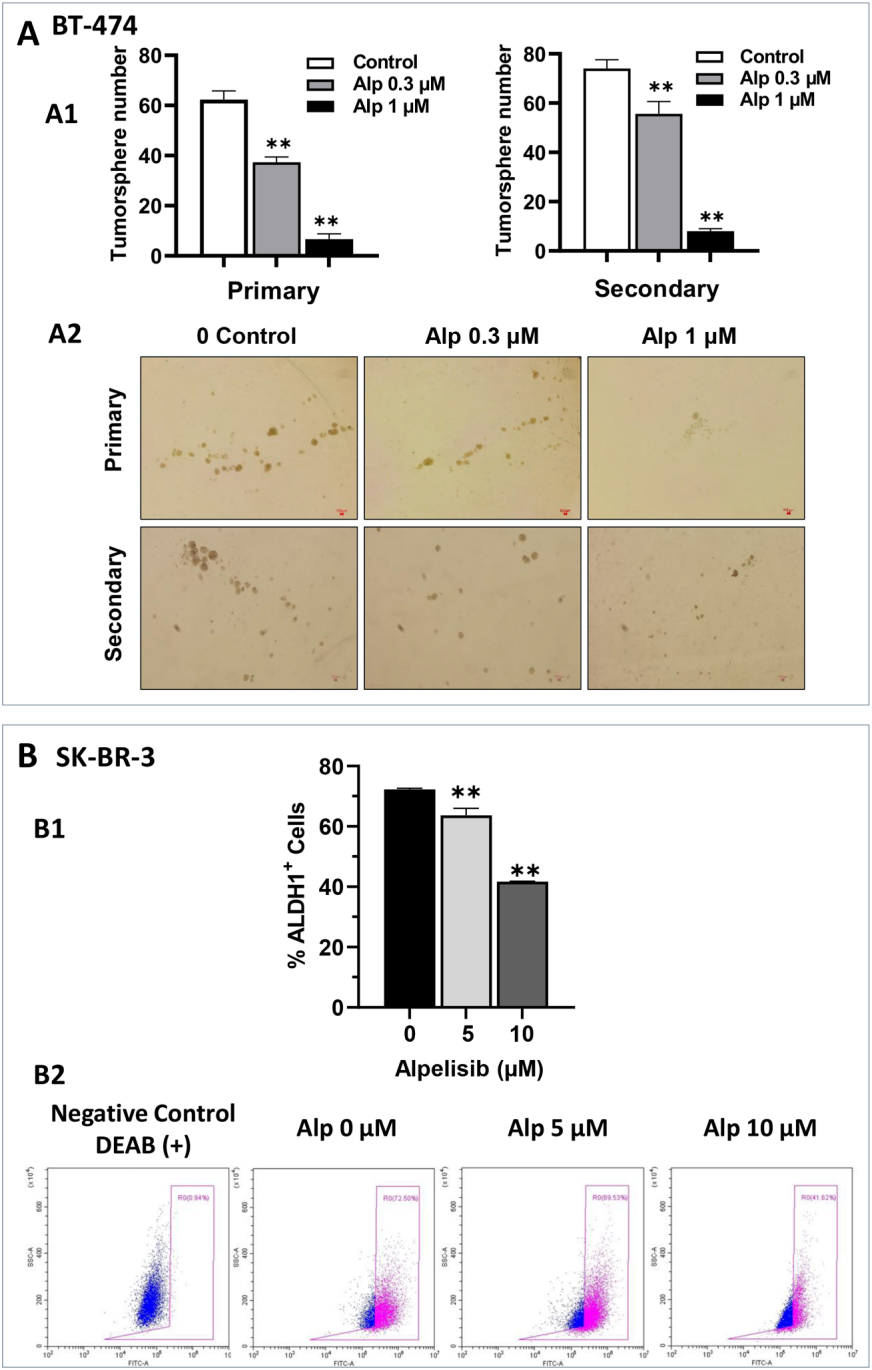
Alpelisib inhibits stemness activity in both SK-BR-3 and BT-474 cells. **(A)** Effect of alpelisib on tumor sphere formation in BT-474 cells. A1. BT-474 cells were seeded in low-attachment 24-well plates at a density of 1, 000 cells/well and treated with various concentrations of alpelisib (0, 0.3, 1 μM) for 7 days. Spheres > 40 μm in diameter were counted and statistically analyzed. Secondary sphere formation was assessed using single cells derived from primary spheres after trypsinization. The experiments were performed in triplicate. The representative images of primary tumorspheres are shown in A2. ** p < 0.01 (indicated group vs. the untreated control). **(B)** Effect of alpelisib on ALDH1 activity in SK-BR-3 cells. B1. SK-BR-3 cells were treated with different concentrations of alpelisib (0, 5, 10 μM) for 3 days. ALDH1 activity was measured with the ALDEFLUOR assay using flow cytometry. Representative plots of the ALDH1+ cell gating are shown in B2, and the percentages of ALDH1+ cells are quantified using one-way ANOVA followed by Tukey’s *post-hoc* test for multiple comparisons (** p < 0.01) (indicated group vs. the untreated control).

Given the critical role of cancer stem cells in tumor development, progression, and drug resistance (31), we evaluated the effect of alpelisib on the stem-like properties of BT-474 and SK-BR-3 cells using cell-line-specific assays. Tumorsphere formation assays, a classical method for studying the stem-like properties of BT-474 cells, were employed to assess the impact of alpelisib for this cell line. As shown in **Figure 2A**, alpelisib significantly inhibited both primary and secondary tumorsphere formation. Notably, the reduction in secondary sphere formation reflects a marked decrease in cancer stem cell self-renewal capacity, demonstrating a potent anti-stemness effect. SK-BR-3 cells are known to have limitations in forming typical tumorspheres in standard assays (32). In contrast, the ALDEFLUOR assay, which measures ALDH1 activity as a surrogate marker for cancer stem cells (33), is commonly used for SK-BR-3 cells. Therefore, we employed this assay to quantify the stemness in SK-BR-3 cells. The results from the ALDEFLUOR assays on alpelisib-treated SK-BR-3 cells indicate that alpelisib treatment led to a significant reduction in the percentage of ALDH1+ cells (**Figure 2B**), further validating its inhibitory effect on cancer stemness in SK-BR-3 cells. Together, these findings demonstrate that alpelisib effectively suppresses cancer stemness in both SK-BR-3 and BT-474 cells, highlighting its potential as a therapeutic agent targeting cancer stem cell populations.

### Metformin synergistically enhances alpelisib-induced inhibition of proliferation and colony formation in SK-BR-3 and BT-474 cells

**Figure 3:**
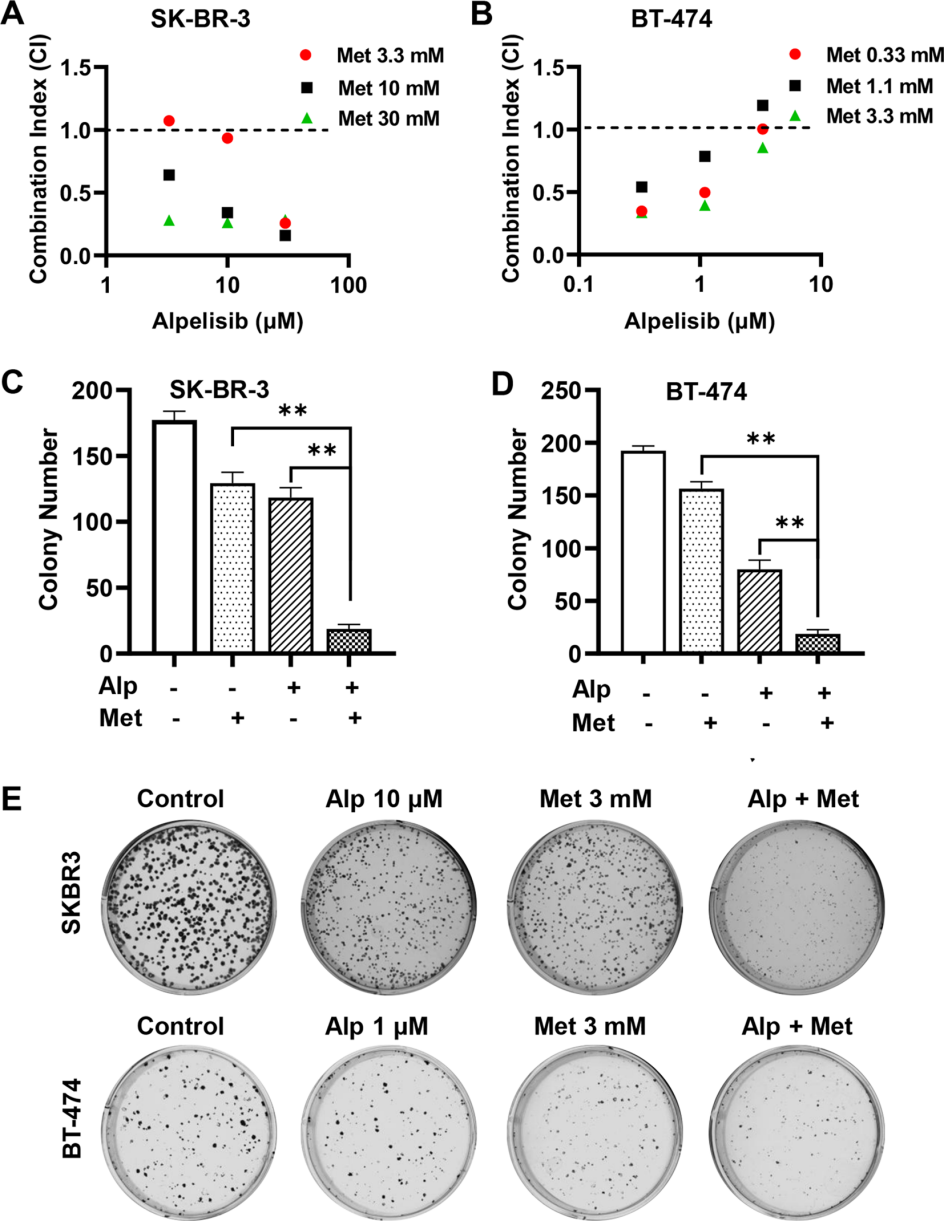
Metformin synergistically enhances alpelisib-induced inhibition of proliferation and colony formation in SK-BR-3 and BT-474 cells. **A** and **(B)** Combination index (CI) analysis of SK-BR-3 **(A)** and BT-474 **(B)** cells treated with alpelisib (Alp) and metformin in various combinations. Cells were treated with the indicated drug concentrations for 5 days, and survival fractions were measured using CCK-8 assays. CI values were calculated using CompuSyn software based on the Chou-Talalay method. C & **(D)** Clonogenic assays of SK-BR-3 **(C)** and BT-474 **(D)** cells treated with alpelisib, metformin, or their combination at the indicated concentrations for 14 days. Colonies were stained with crystal violet and quantified. ** p < 0.01 (combination vs. single-agent). **(E)** Representative images of clonogenic assays for SK-BR-3 and BT-474 cells with indicated treatments.

Previously, we demonstrated that metformin, a first-line drug for diabetes, inhibits the proliferation and stemness of erbB2-overexpressing breast cancer cells (34). Our preliminary studies also showed that metformin effectively inhibited the proliferation of SK-BR-3 and BT-474 cells, which was associated with the phosphorylation and activation of AMPK (**Supplementary Figure 1**). Since a major side effect of alpelisib is a significant increase in blood sugar levels (10, 11), testing the effect of the alpelisib-metformin combination on these cancer cells represents a rational therapeutic strategy. We then evaluated the combination effect on the proliferation of SK-BR-3 and BT-474 cells using the CCK-8 assay, followed by synergistic analysis with the combination index (CI) using the Chou-Talalay method. In this experiment, SK-BR-3 cells were treated with 0, 3.3, 10, and 30 μM of alpelisib alone or in combination with 0, 3.3, 10, and 30 mM of metformin. BT-474 cells were treated with 0, 0.33, 1, and 3 μM of alpelisib alone or in combination with 0, 0.33, 1.1, and 3.3 mM of metformin. As shown in **Figures 3A, B** most of the CI values between alpelisib and metformin for each cell line were below 1.0, with the exception of one combination ratio in each cell line. These data suggest a synergistic activity of alpelisib and metformin in inhibiting proliferation in these cells. To test the potential synergistic effect of the metformin-alpelisib combination on the colony formation efficiency of breast cancer cells, SK-BR-3 cells were treated with 10 μM of alpelisib, 3 mM of metformin, or their combination, while BT-474 cells were treated with 1 μM of alpelisib, 3 mM of metformin, or their combination for 10 days, followed by colony quantification and analysis (**Figures 3C-E**). While each drug alone was able to inhibit colony formation, the combination of metformin with alpelisib resulted in a significant reduction in colony formation in both cell lines, indicating the enhanced efficacy of this combination.

**Figure 4:**
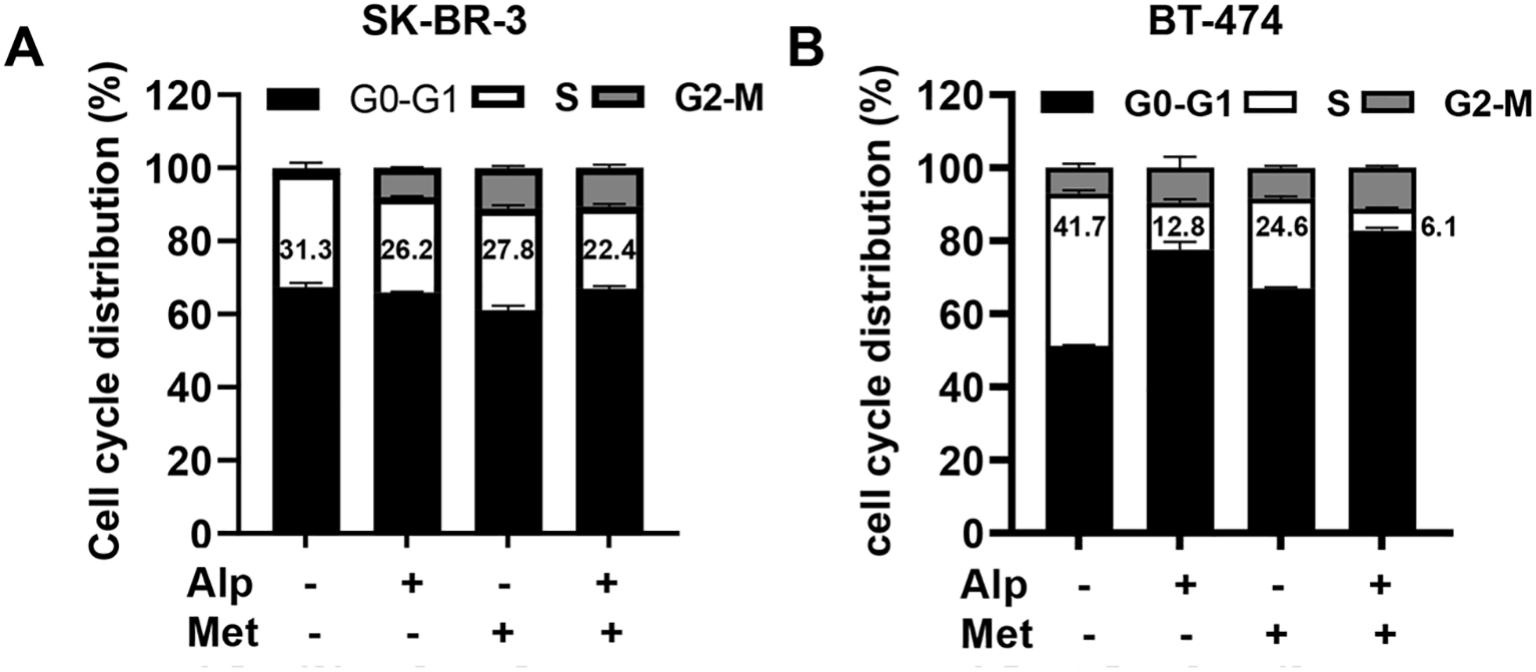
Combination of alpelisib and metformin enhances cell cycle arrest in SK-BR-3 and BT-474 cells. **(A)** SK-BR-3 cells were treated with 3 μM alpelisib, 5 mM metformin or their combination for 24 hours. **(B)** BT-474 cells were treated with 0.33 μM alpelisib, 1 mM metformin, or their combination for 24 hours. Treated cells were harvested and analyzed for cell cycle distribution using flow cytometry in triplicate. The percentages of cells in each phase of the cell cycle were calculated using MODFIT software, with the percentages of cells in the S phase highlighted.

Moreover, we examined the effect of the metformin-alpelisib combination on cell cycle progression in SK-BR-3 and BT-474 cells (**Figure 4**). To this end, SK-BR-3 cells were treated with 3 μM alpelisib, 5 mM metformin, or their combination, while BT-474 cells were treated with 0.33 μM alpelisib, 1 mM metformin, or their combination for 24 hours. Cell cycle analysis indicates that the combination of metformin and alpelisib induces enhanced inhibition of cell cycle progression in both cell lines, as evidenced by a significant reduction in the percentage of cells in the S phase compared to the results with the corresponding single agents. Taken together, these results demonstrate that the combination of metformin and alpelisib induces strong synergistic effects on the proliferation and colony formation of SK-BR-3 and BT-474 cells.

### Metformin-alpelisib combination induces enhanced inhibition of cancer cell stemness in SK-BR-3 and BT-474 cells

**Figure 5:**
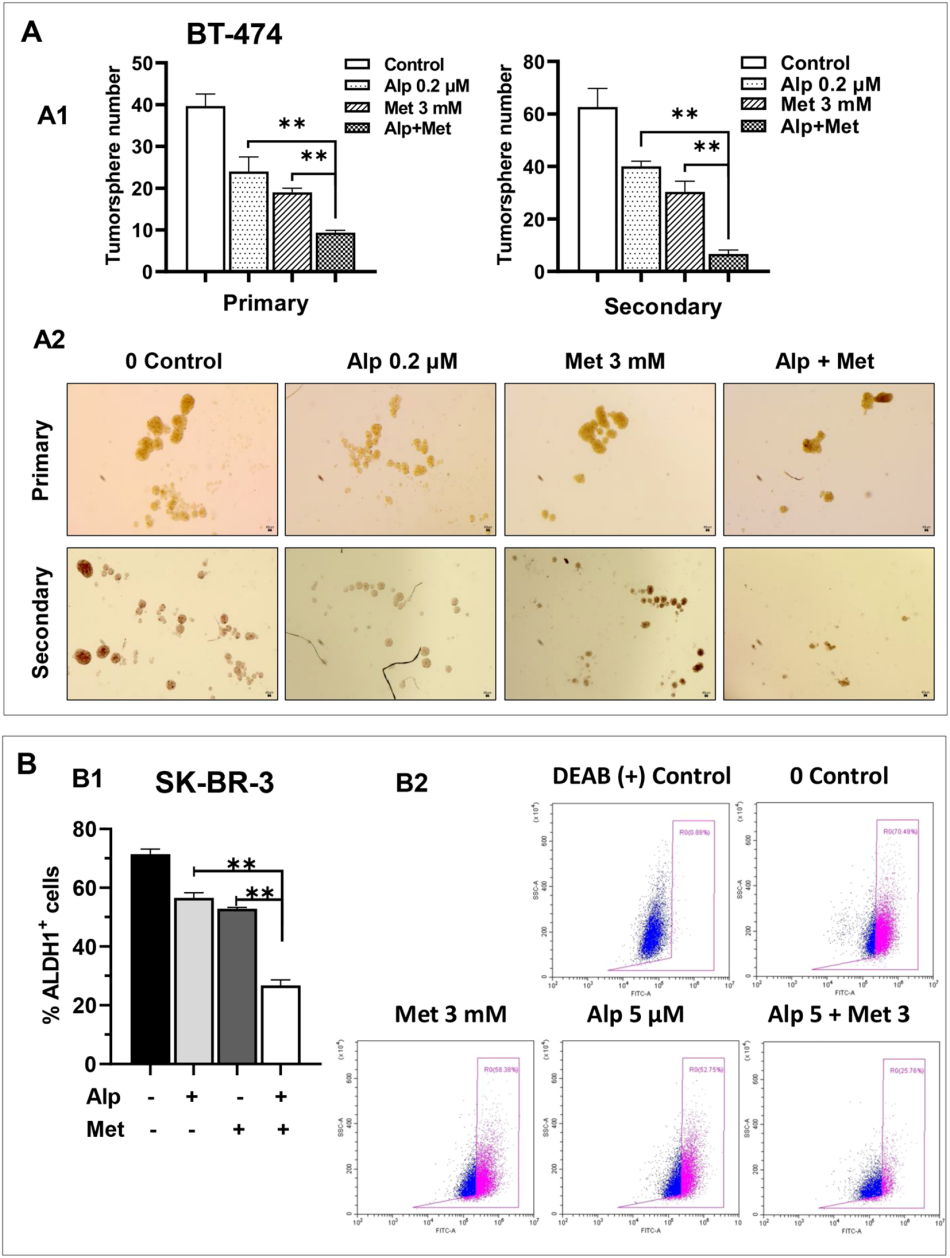
Metformin enhances alpelisib-induced inhibition of stemness in SK-BR-3 and BT-474 cells. **(A)** Effect of alpelisib and metformin on tumor sphere formation in BT-474 cells. A1. For primary sphere formation, BT-474 cells were seeded in low-attachment 24-well plates at 1, 000 cells/well and treated with 0.2 μM alpelisib, 3 mM metformin, or their combination for 7 days. Spheres >25 μm in diameter were counted and statistically analyzed. Secondary sphere formation was assessed using single cells derived from primary spheres after trypsinization. The experiments were performed in triplicate. ** p < 0.01 (combination vs. single-agent). A2. Representative images of primary and secondary spheres as described in A1. **(B)** Effect of alpelisib and metformin on ALDH1 activity in SK-BR-3 cells. B1. SK-BR-3 cells were treated with 5 μM alpelisib, 3 mM metformin, or their combination for 48h. ALDH1 activity was analyzed using the ALDEFLUOR assay with flow cytometry. Based on DEAB-treated negative controls, ALDH1+ cell populations were quantified. Data in triplicate were analyzed using one-way ANOVA followed by Tukey’s *post-hoc* test for multiple comparisons (** p < 0.01) (combination vs. single-agent). B2. Representative flow cytometry histograms of ALDEFLUOR assays described in B1.

**Figure 6:**
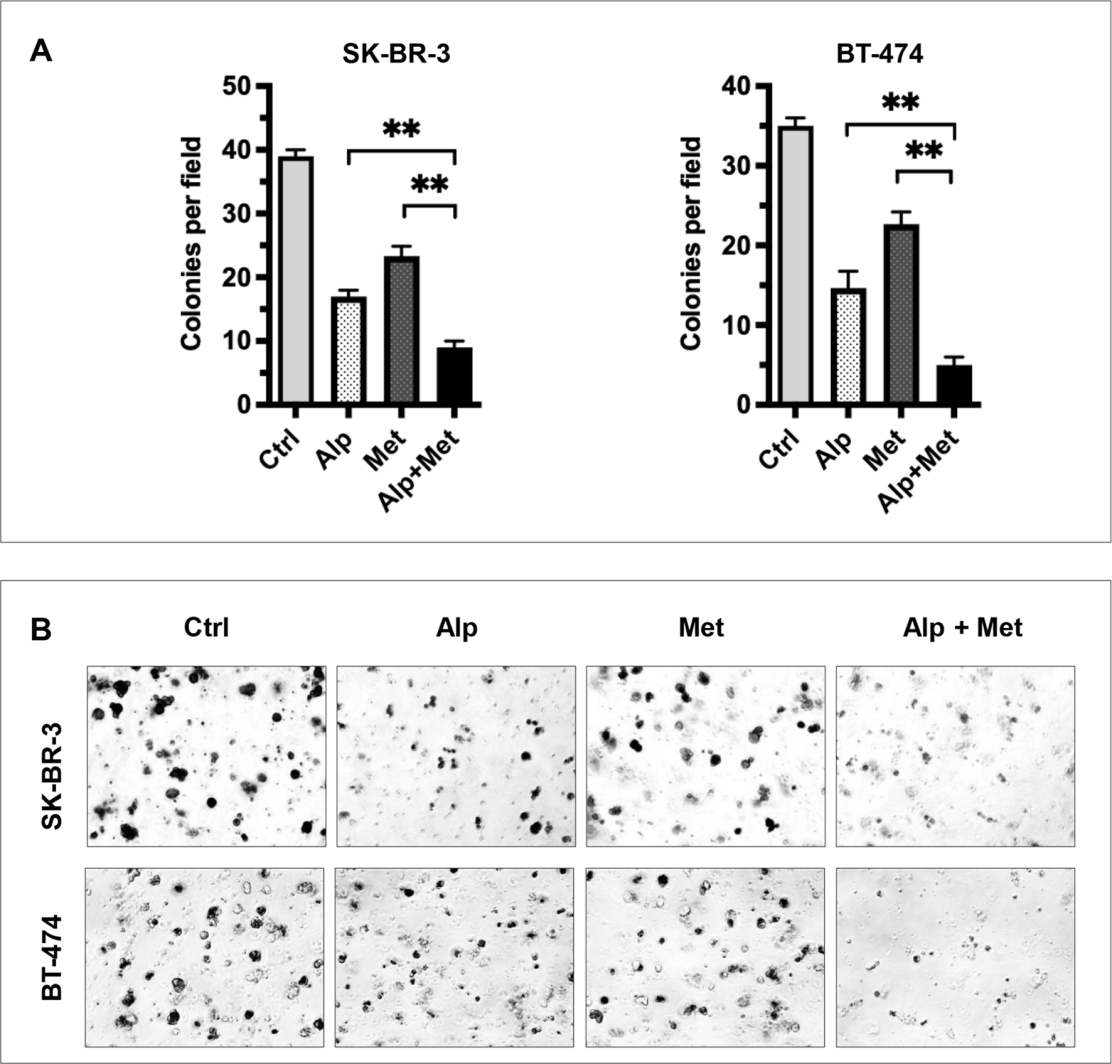
Metformin enhances alpelisib-induced inhibition of anchorage-independent growth in SK-BR-3 and BT-474 cells. SK-BR-3 and BT-474 cells were treated with 2 μM alpelisib (Alp), 3 mM metformin (Met), or their combination (Alp + Met) in 3D Matrigel culture for 14 days. **(A)** Quantified colony counts. Colonies with a diameter ≥25 μm were counted, and data are presented as the mean ± SD from three independent experiments. Statistical significance was determined by one-way ANOVA with Tukey’s *post hoc* test (**p < 0.01, combination vs. single-agent). **(B)** Representative images of 3D colonies in each cell line under the indicated treatment conditions.

Given that both alpelisib and metformin may inhibit cancer cell stemness, we next determined the effect of the metformin-alpelisib combination on the stemness of SK-BR-3 and BT-474 cells. Similar to the experiment in **Figure 2**, we evaluated this effect on BT-474 cells using tumorsphere assays. As shown in **Figure 5A**, the combination of 3 mM metformin and 0.2 μM alpelisib induced a significant reduction in both primary and secondary tumorspheres in each cell line, compared to the results with the single agents. These results suggest that the combination enhances the inhibition of stemness in BT-474 cells. To evaluate the effect of the combination on the stemness of SK-BR-3 cells, the cells treated with 5 μM alpelisib and 3 mM metformin, either alone or in combination, were analyzed using the ALDEFLUOR assay (**Figure 5B**). The results showed that the percentage of ALDH1+ cells in the combination group was significantly decreased compared to the single agent groups. To further demonstrate that metformin-alpelisib combination induces enhanced stemness inhibition, we evaluated anchorage-independent growth, a key property of cancer stem cells (35), using 3D culture in semisolid Matrigel under low growth factor conditions. As shown in **Figure 6**, single-agent treatments reduced 3D colony formation in both cell lines. However, the combination of metformin and alpelisib resulted in significantly enhanced inhibition compared to monotherapies. Together, these results from tumorsphere, ALDEFLUOR, and anchorage-independent growth assays demonstrate that the metformin-alpelisib combination synergistically inhibits cancer stemness in HER2+ breast cancer cells.

### Metformin enhances alpelisib-induced inhibition of receptor tyrosine kinase signaling and stemness markers in SK-BR-3 and BT-474 cells

**Figure 7:**
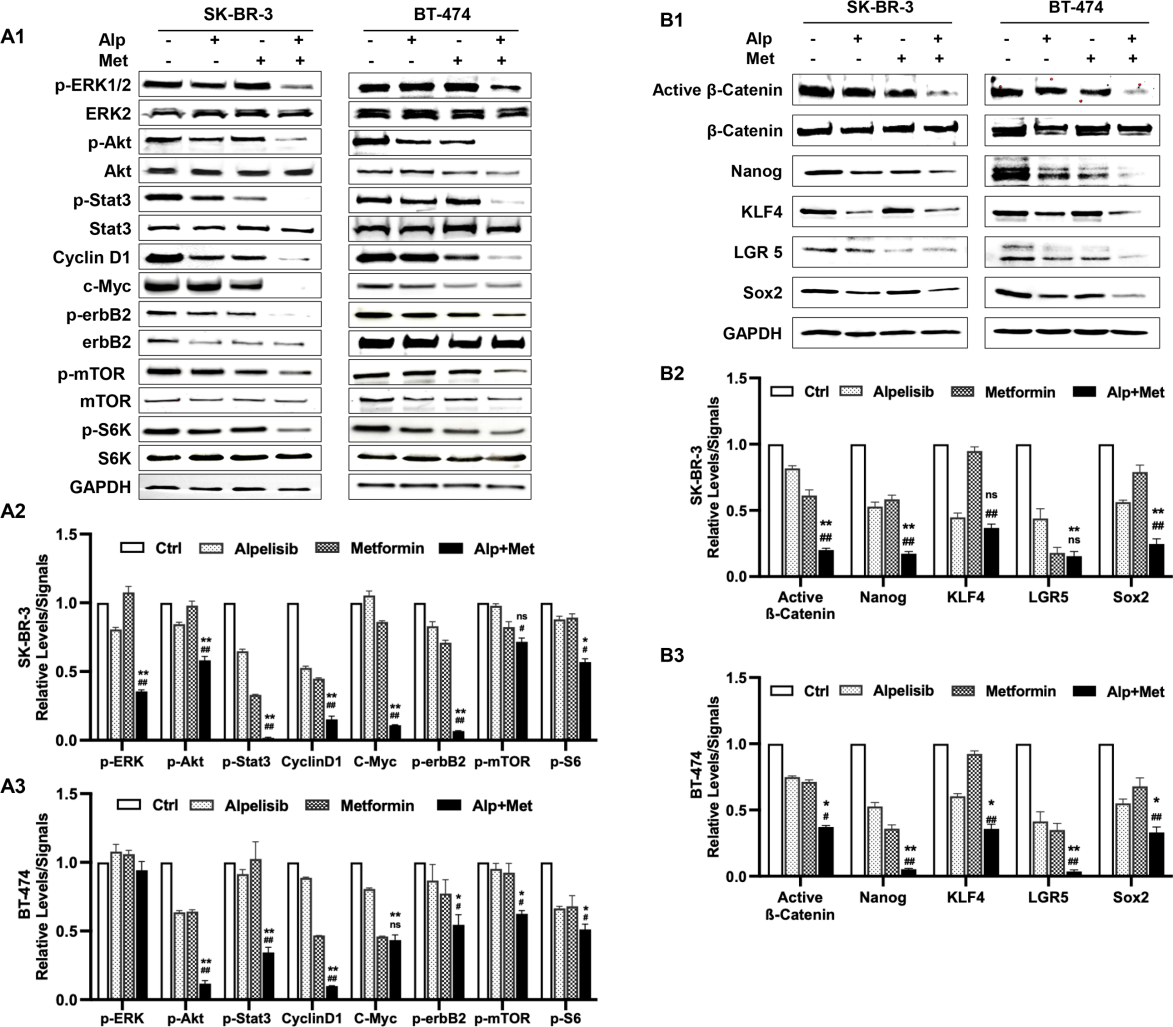
Metformin enhances alpelisib-induced inhibition of receptor tyrosine kinase signaling and stemness regulators in SK-BR-3 and BT-474 cells. SK-BR-3 cells were treated with 3.3 μM alpelisib, 5 mM metformin, or their combination for 48 hours. BT-474 cells were treated with 1.1 μM alpelisib, 1.1 mM metformin, or their combination for 48 hours. The treated cells were collected for protein extraction, followed by Western blot analysis to detect the relative levels of total or phosphorylated (p-) proteins of the indicated markers. **(A)** Effects of the alpelisib–metformin combination on receptor tyrosine kinase (RTK) pathway components (ERK, Akt, Stat3, Cyclin D1, erbB2, mTOR, S6K). **(B)** Effects of the alpelisib–metformin combination on the expression/activation of key stemness markers (β-catenin, Nanog, KLF4, LGR5, Sox2). A2/A3 and B2/B3 show the quantified data derived from A1 and B1, respectively. For quantification in each panel, Western blot band intensities were quantified using ImageJ and normalized to the indicated loading controls. Phosphorylated protein levels were further normalized to the corresponding total protein levels. Quantified data represent the mean of three independent experiments. */^#^ p < 0.05; **/^##^ p < 0.01; ns, not significant. */** Combination (Alp + Met) vs. alpelisib; ^#^/^##^ Combination vs. metformin.

To investigate the mechanism underlying the enhanced anti-tumor activity induced by the metformin-alpelisib combination, we analyzed key markers of the receptor tyrosine kinase (RTK) pathway and related oncogenic signals, followed by examination of stemness-related pathways. SK-BR-3 cells were treated with 3.3 μM alpelisib and 5 mM metformin, either alone or in combination, while BT-474 cells were treated with 1.1 μM alpelisib and 1.1 mM metformin, either alone or in combination. We examined critical effectors downstream of PI3K—Akt, mTOR, and S6K—which mediate survival and growth signals (36). Additionally, we evaluated ERK and STAT3, key regulators in the MAPK/ERK and JAK/STAT pathways respectively, both of which can cross-talk with the PI3K/Akt pathway (37, 38). Cyclin D1 and c-Myc, key factors downstream of multiple signaling pathways critical for regulating cell cycle and growth (39, 40), were also analyzed. Although erbB2 is an upstream RTK, its activation status was assessed given its regulation through interactions and feedback loops. As shown in **Figure 7A**, results from both cell lines demonstrated that the combination treatment induced enhanced inhibition of signaling in these pathways, as indicated by reduced protein levels of phosphorylated or active forms of the individual markers. The combination-induced suppression was particularly prominent for p-Akt, p-Stat3, c-Myc, p-erbB2, and p-ERK1/2. These results suggest that the metformin-alpelisib combination not only induces enhanced inhibition of signaling downstream of the PI3K pathway but also exerts a broader impact on the RTK network.

To further elucidate the mechanisms underlying the enhanced inhibition of cancer stemness, we investigated the effect of this regimen on stemness-associated signaling pathways and transcription factors. Beyond the Wnt/β-catenin pathway, a well-established regulator of cancer stemness (41), we analyzed the expression of core pluripotency factors, including Nanog, KLF4, LGR5, and Sox2, which are critical for maintaining self-renewal, tumorigenicity, and therapy resistance in breast cancer stem cells (42–45). As shown in **Figure 7B**, Western blot analysis revealed that both metformin and alpelisib monotherapies reduced the expression of active β-catenin and these stemness-related markers to varying degrees in both cell lines. Notably, the combination treatment resulted in markedly enhanced suppression of these factors compared to either agent alone. Specifically, in SK-BR-3 cells, the combination significantly decreased protein levels of active β-catenin, Nanog, KLF4, LGR5, and Sox2. A similar enhanced inhibitory trend was observed in BT-474 cells, particularly for active β-catenin, Nanog, and LGR5, although some cell line-specific variations in response were noted. Together with the c-Myc inhibition observed in **Figure 7A**, these findings indicate that the metformin-alpelisib combination synergistically targets both oncogenic signaling networks and stemness regulatory pathways, providing an integrated mechanistic basis for its enhanced anti-tumor effects.

## Discussion

This study was designed to test the effect of alpelisib on erbB2-overexpressing breast cancer and the potential synergetic effect of combining metformin with alpelisib therapeutic regimen. Our study demonstrates that alpelisib, a PI3K inhibitor, effectively inhibits the proliferation, colony formation, and cancer stemness of erbB2-overexpressing breast cancer cells. Furthermore, we show that metformin synergistically enhances these effects. The combination of alpelisib and metformin significantly suppresses critical signaling pathways, including PI3K/AKT/mTOR and RTK networks, as well as Wnt/β-catenin signaling and core stem cells regulators. These findings underscore the potential of combining alpelisib with metformin to enhance therapeutic efficacy against erbB2-overexpressing breast cancer.

Alpelisib is primarily recognized as an effective treatment for patients with HR+/HER2- breast cancer who harbor PIK3CA mutations (46). However, emerging evidence suggests that its utilization could provide substantial benefits for HER2+ breast cancer patients as well (18). Although clinical trials are ongoing to explore alpelisib’s safety and efficacy as an adjunct to standard HER2-targeted therapies (15, 18), additional preclinical studies in this area will facilitate the broader application of alpelisib in HER2+ breast cancer. Our results from tests of alpelisib as a single agent on two HER2+ breast cancer cell lines provide fundamental evidence supporting its potential use in HER2+ breast cancer. In particular, we demonstrated that metformin significantly enhances the efficacy of alpelisib in PIK3CA wild-type SK-BR-3 cells. This suggests that the combination of metformin with alpelisib may expand therapeutic options, especially for HER2+ patients without PIK3CA mutations. It could also mitigate alpelisib-induced hyperglycemia to improve tolerability and enable the administration of more effective doses for better outcomes. The differential sensitivity of BT-474 and SK-BR-3 cells to alpelisib was an interesting finding in this study. Specifically, BT-474 cells demonstrated greater sensitivity to alpelisib (IC_50_ = 0.329 μM) compared to SK-BR-3 cells (IC_50_ = 8.022 μM). This variability likely reflects the heterogeneity of HER2+ breast cancer, in which cell lines of the same subtype often exhibit distinct molecular profiles and drug sensitivities. The underlying mechanism for this disparity may be partially linked to the PIK3CA mutation status of each cell line. BT-474 cells, which harbor the PIK3CA-K111N mutation (47), exhibit hyperactivation of the PI3K/AKT/mTOR pathway, rendering them highly dependent on this signaling axis for survival and proliferation. Conversely, SK-BR-3 cells, derived from a metastatic tumor, lack PIK3CA mutations (48). The signaling in these cells may be predominantly driven by HER2 RTK activity, which may activate compensatory pathways to support cell growth. Although PIK3CA mutation status appears to play a role in these differential responses, the reported lower sensitivity of SK-BR-3 cells to other anticancer drugs suggests a more intricate signaling network in these cells (49, 50). Interestingly, the combination of metformin with alpelisib showed a synergistic effect in both cell lines, with a more pronounced effect observed in BT-474 cells. This heightened synergy in BT-474 cells is likely attributable to metformin’s dual action: inhibiting PI3K/mTOR (through AMPK activation) and mitochondrial complex I, thereby amplifying metabolic stress in cells harboring PIK3CA mutations. Further research is warranted to fully understand the mechanisms underlying the differential responses between these two cell lines. Given the heterogeneity of HER2+ breast cancer, testing additional cell line models may provide a more in-depth understanding of the underlying mechanisms.

Hyperglycemia is a significant side effect associated with alpelisib, occurring in approximately 60% of patients treated with this drug (10). This adverse effect may confer a survival advantage to cancer cells and diminish therapeutic efficacy (9). The combination of metformin, a well-known anti-diabetic agent (51), with alpelisib offers a proactive management strategy to mitigate alpelisib-associated hyperglycemia (25). During the course of our project, a recent report from the METALLICA trial showed that the prophylactic use of metformin demonstrated significant benefits compared to historical data, as indicated by a markedly lower incidence of hyperglycemia (27). In contrast to this report, which focuses on managing of hyperglycemia side effects, our results provide additional evidence of the synergistic anti-tumor effect induced by the metformin-alpelisib combination. Specifically, metformin enhances alpelisib’s suppression of oncogenic and stemness networks. Thus, while the METALLICA trial highlights metformin’s value as a prophylactic agent to improve tolerability, our findings indicate that it also contributes directly to anti-tumor efficacy. Together, these complementary benefits indicate that incorporating metformin into alpelisib-based regimens is not merely supportive but represents a rational strategy to both prevent treatment-limiting hyperglycemia and enhance cancer control. This dual action could significantly improve the therapeutic index of alpelisib, particularly in HER2+ breast cancer.

The enhanced inhibition of cancer cell stemness by the metformin-alpelisib combination is particularly notable. Cancer stem cells play a crucial role in tumor recurrence, metastasis, and resistance to therapy (31). Our results demonstrate that the combination therapy not only reduces conventional cancer stem cell markers, including ALDH1 activity, and diminishes tumorsphere formation in both primary and secondary generations, but also significantly suppresses anchorage-independent growth in 3D culture (**Figure 6**), a functional hallmark of stemness and malignant potential (35). The ability to disrupt anchorage-independent growth further suggests the potential of this combination to inhibit metastatic potential and therapeutic resistance. This consistent inhibition across multiple complementary assays (ALDEFLUOR, tumorsphere, and 3D Matrigel culture) provides compelling evidence that the combination therapy effectively targets the self-renewal capacity and clonogenic survival of cancer stem cells. Taken together, these observations indicate that dual targeting of both bulk tumor cell proliferation and cancer stemness by this combination therapy could lead to more durable treatment responses and improved clinical outcomes, particularly for aggressive and therapy-resistant HER2+ breast cancer subtypes.

Our mechanistic studies focused on RTK signaling and stem cell regulatory networks. Metformin enhanced the inhibitory effects of alpelisib on key PI3K effectors, including phosphorylated Akt, mTOR, cyclin D1, and S6K (3), and further suppressed oncogenic regulators such as p-ERK and p-STAT3. The combination also reduced activity of Wnt/β-catenin signaling and pluripotency factors including c-Myc, Nanog, KLF4, LGR5, and Sox2 (**Figures 7A, B**), consistent with reduced stemness phenotypes observed in **Figure 5** and **Figure 6**. These results suggest that synergy arises from simultaneous metabolic stress induced by metformin and PI3K pathway inhibition by alpelisib, converging to disrupt cancer cell survival and stemness. Broad suppression of these oncogenic and stem cell pathways may help overcome compensatory mechanisms that limit single-agent efficacy (39, 40).

We also evaluated the activation of AMPK, a key downstream target of metformin (52). Metformin induced AMPK phosphorylation in both cell lines (**Supplementary Figure 1**), but the combination did not further enhance AMPK activation (data not shown). These findings indicate that synergy may occur downstream of AMPK activation, likely through interactions with RTK-related pathways. Further studies are warranted to define the precise mechanisms. Our study lays a solid foundation for further clinical investigations into the combination of metformin and alpelisib for the treatment of erbB2-overexpressing breast cancer. It also provides a framework for exploring similar combination strategies to address the limitations of targeted therapies, including resistance and adverse metabolic effects. However, additional preclinical and clinical studies are necessary to optimize the dosing strategies and fully characterize the long-term benefits and potential risks associated with this combination.

In conclusion, the results from this study demonstrate that alpelisib offers potent anti-tumor activity in erbB2/HER2-overexpressing breast cancer, which can be significantly enhanced by combining it with metformin. The synergistic interaction between alpelisib and metformin underscores a promising therapeutic approach for this breast cancer subtype. Metformin’s ability to both enhance anti-tumor efficacy and mitigate metabolic side effects highlights its potential to broaden the clinical applicability of alpelisib, paving the way for improved patient outcomes in this challenging cancer subtype.

## Data Availability

The raw data supporting the conclusions of this article will be made available by the authors, without undue reservation.
