# Supplementary figures and images for: Metformin enhances alpelisib sensitivity in HER2+ breast cancer by suppressing cancer stemness and oncogenic signaling
Source: Front Oncol. 2025 Nov 19;15:1631415. doi: 10.3389/fonc.2025.1631415 (PMC12672257; doi:10.3389/fonc.2025.1631415)

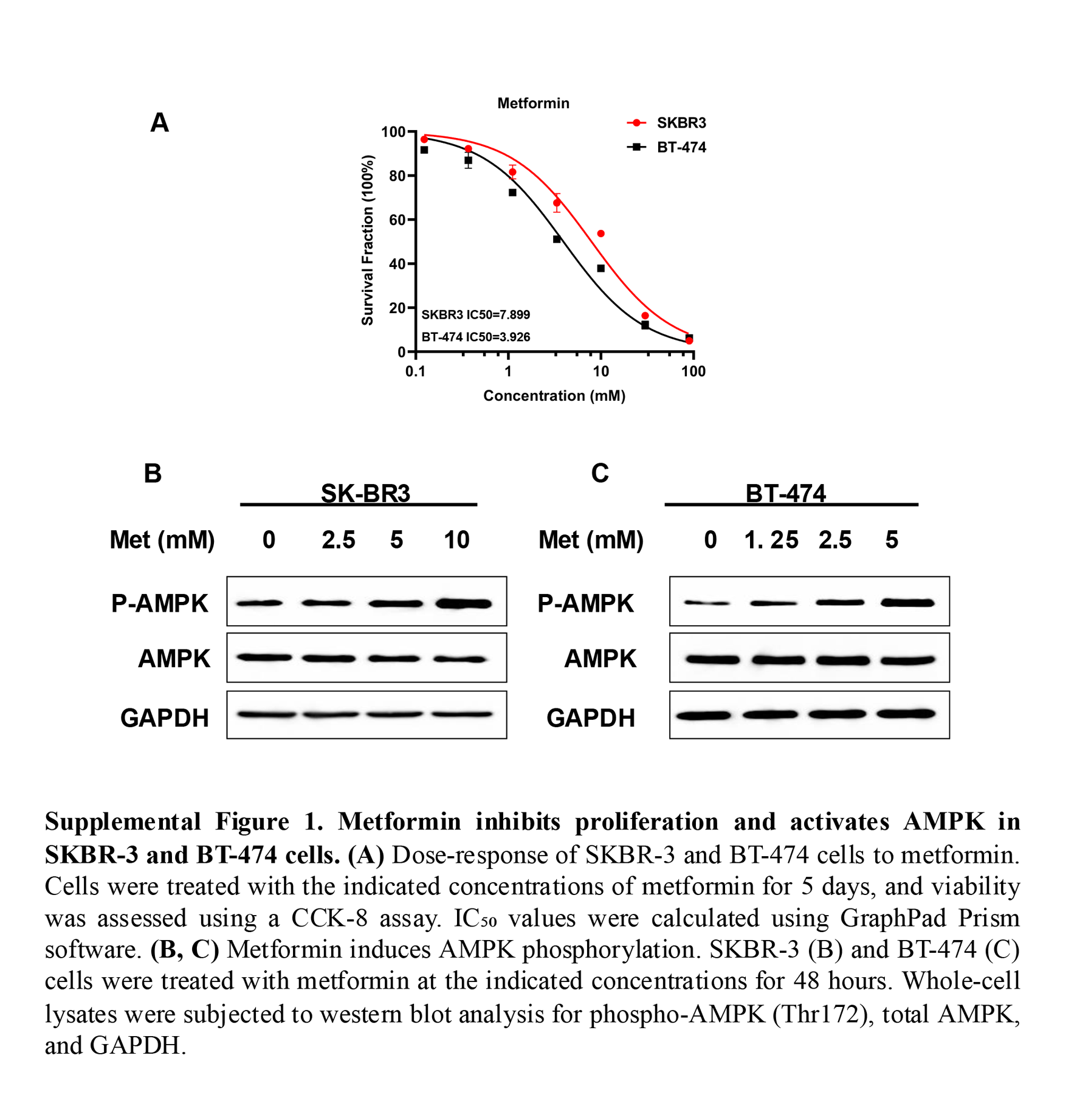

Supplement: Supplementary file 1 [file Image1.tif]
